# A spark of change: developing an innovative gerontological nursing intervention mapping initiative for training and education (IGNITE)

**DOI:** 10.1186/s12909-024-05240-5

**Published:** 2024-03-08

**Authors:** HeeKyung Chang, Young Joo Do

**Affiliations:** 1 College of Nursing, Gerontological Health Research Center in Institute of Health Sciences, Gyeongsang National University, 52727, 816-15, Jinju-daero, Jinju, South Korea; 2 College of Nursing, Gyeongsang National University, 52727, 816-15, Jinju-daero, Jinju, Gyeongnam South Korea

**Keywords:** Gerontological nursing, Intervention mapping approach, Nursing education, Professional nurses, Transformative learning theory

## Abstract

**Background:**

With an aging global population and advancements in medical technology, there is an urgent need for innovative gerontological nursing education programs. This study aimed to develop and evaluate the Innovative Gerontological Nursing Intervention Mapping Initiative for Training and Education (IGNITE) program. This program is a digital platform-based postgraduate nursing curriculum that employs the Intervention Mapping Approach (IMA) and Transformative Learning Theory to address the evolving needs of gerontological nursing.

**Methods:**

The IGNITE program’s development process encompassed a comprehensive approach, including needs assessment, mapping of course objectives, integration of theory-based methods and strategies, course design, implementation, and rigorous evaluation. The pilot evaluation study involved pre- and post-tests focused on ageism, attitudes towards elder care, knowledge about older adults, transformative behavior change, and program satisfaction. The findings revealed significant improvements across all these dimensions, affirming the effectiveness of the program.

**Results:**

The program leveraged experiential learning, critical reflection, and rational discourse to facilitate transformative educational experiences. Notably, pre- and post-test comparisons showed marked improvements in attitudes towards older adult care and dementia care knowledge. Participants expressed high satisfaction with the program, with significant reported changes in transformative behaviors. The study also illuminated the initial negative attitudes of clinical nurses towards older adults and underscored the importance of transformative learning experiences in fostering empathy and understanding.

**Conclusions:**

The IGNITE program lays a foundational framework for developing educational materials that promote transformative learning and self-reflection among healthcare professionals. This approach can lead to innovative nursing practices and personal growth. The application of the IMA and Transformative Learning Theory in gerontological nursing education shows significant promise. Future research should focus on exploring the long-term impacts of such programs and their applicability in diverse healthcare settings.

## Background

As Korea rapidly approaches the threshold of a super-aging society, with projections indicating that 20.6% of its population will be older adults by 2025 [1], there is an emerging critical need for an adaptive healthcare service and delivery system. This evolution is tailored to the changing demographic landscape, aiming to effectively manage chronic diseases and incorporate advanced medical technologies [2]. In response, the Korean government has strategized to enhance wellness, well-dying, and well-aging [3]. This paradigm shift accentuates the importance of gerontological nursing education, particularly focusing on the emotional and creative dimensions of older adult care, incorporating empathy and the application of smart healthcare technologies, such as digital platforms and assistive devices.

The Intervention Mapping Approach (IMA), traditionally employed in health promotion, has been adapted for the development of training and educational programs for practicing nurses [4–9]. IMA is a systematic process that includes a thorough needs assessment, setting objectives, selecting theory-based methods, and program implementation and evaluation. Despite IMA’s proven effectiveness in program development, there is a notable gap in research on post-development program evaluation, particularly in Korea and other regions employing IMA [10, 11]. This gap is significant, considering the need for comprehensive assessments of the effectiveness of IMA programs, especially since most existing studies have focused primarily on development stages or protocol outlines. Previous research has shown IMA’s potential to effectuate changes in cognitive, attitudinal, belief, and self-efficacy aspects among practicing nurses [12, 13].

In the current healthcare paradigm, which is shifting towards precision and predictive medicine, gerontological care is increasingly focusing on personalized, patient-centered management. This is crucial considering the prevalence of multiple chronic diseases among older adults and their diverse physical, functional, and cognitive needs [14]. Standardized nursing protocols may be insufficient for such varied requirements. Therefore, specialized gerontological nursing education, aligned with the advancements of the Fourth Industrial Revolution and tailored to the specific needs of older adults, is essential for nurses to effectively engage in gerontological care. The Fourth Industrial Revolution, synonymous with state-of-the-art status, digitization, and smart automation, denotes a paradigm shift in technological advancement. It encompasses the phenomena of hyper-connectivity, super-intelligence, and mega-convergence among people, objects, and spaces, leading to the innovation of industrial structures and the overall societal system. This revolution is underpinned by the technologies of the Internet of Things (IoT), Cyber Physical Systems (CPS), and Artificial Intelligence (AI), including automation, data exchange, and manufacturing technologies. In healthcare, the integration of biotechnology and information and communication technology has enabled the early prediction and management of diseases. Furthermore, the development of personalized medication based on an individual’s genetic characteristics has become feasible, emphasizing the values of ‘prediction,’ ‘customization,’ and ‘personalization.‘ [15].

To transform the approach of experienced nurses in clinical settings and encourage a shift from conventional practices, this study introduces the application of transformative learning theory, fostering a change in cognitive structures through the creation of new meanings. Transformative learning fosters a change in cognitive structures through the creation of new meanings, involving experience, critical reflection, rational discourse, and action [16–18]. Reflective writing, a key element of this theory, facilitates self-communication and empathy, enhancing communication skills, critical thinking, positive coping mechanisms, and empowerment [19, 20]. Building on this, we developed the Innovative Gerontological Nursing Intervention Mapping Initiative for Training and Education (IGNITE) program, a groundbreaking educational program in gerontological care for experienced nurses, utilizing the Intervention Mapping process.

The specific objectives of this study are as follows:

1. To develop the Innovative Gerontological Nursing Intervention Mapping Initiative for Training and Education (IGNITE) program for postgraduate nurses.
2. To evaluate the effectiveness of the IGNITE program
3. To assess the educational outcomes and satisfaction of the participants in the IGNITE program

## Research design and methods

### Design and setting

The IGNITE program employs an educational instructional systems design model, specifically the Intervention Mapping Approach (IMA) [21]. IMA is a systematic process used to develop tailored health interventions, involving a comprehensive assessment of needs, setting measurable objectives, creating strategies, and evaluating outcomes. This program targets professional gerontological nurses in hospitals and clinical nursing graduate students, with a focus on enhancing care for older persons with chronic illness, aligning with the advancements in medical technology and healthcare delivery.

### Methods

**Fig. 1:**
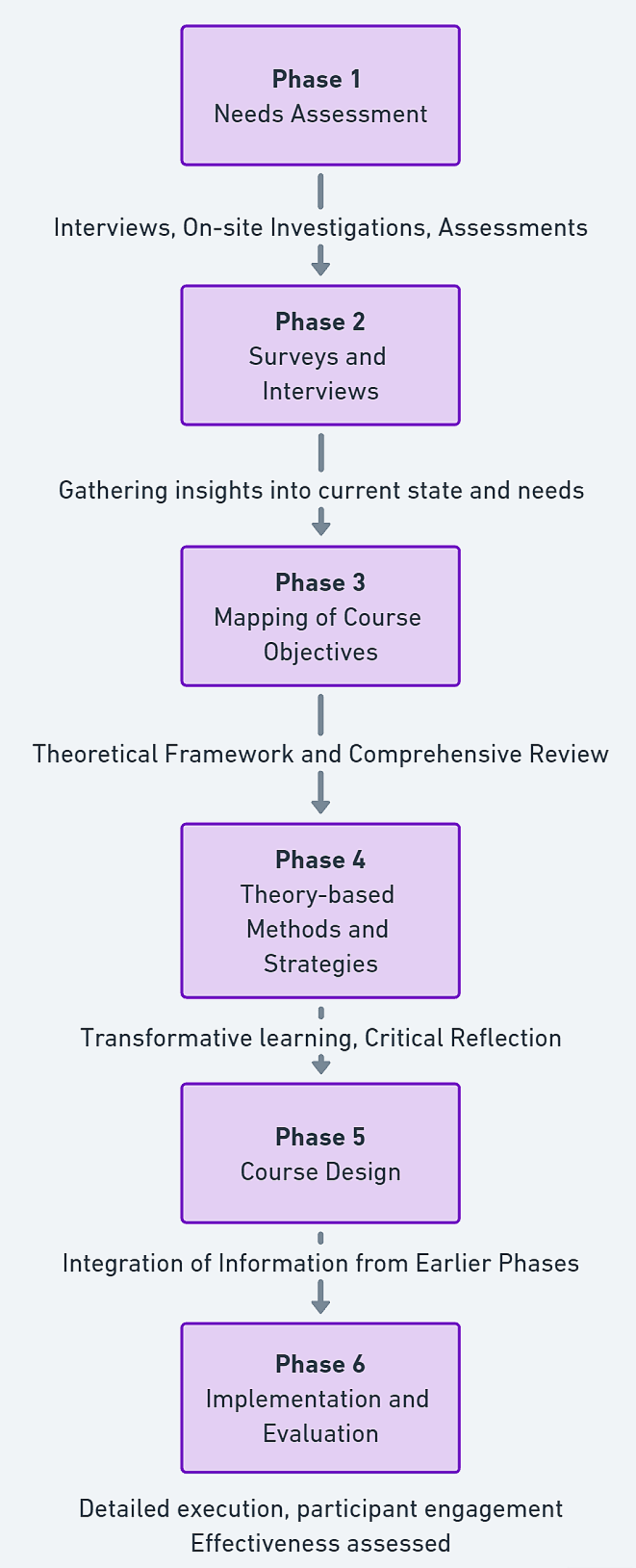
Summary of the IMA phases

The study utilized the Intervention Mapping Protocol as outlined by Bartholomew et al. [21, 22], encompassing six phases (Fig. 1).

#### Needs assessment

This initial phase involved conducting interviews, on-site investigations, and assessments with the target group and relevant stakeholders. A thorough literature review and information gathering were essential to understand the target group’s characteristics, available resources, and potential obstacles for program development. All interview and survey procedures were approved by the Institutional Review Board (IRB) of Gyeongsang National University. Participants provided informed consent, ensuring voluntary participation and confidentiality.

#### Survey and interviews

Conducted at the Gerontological Health Research Center of Gyeongsang National University, these activities gathered insights into the current state and needs of innovative gerontological nursing education. Focus group interviews involved both practicing hospital nurses and nursing students, ensuring a comprehensive needs assessment. These surveys and interviews were integral to the needs assessment phase and formally conducted as part of the research process. The results directly informed the development of the IGNITE program.

#### Mapping of course objectives

The goal was to train nurses in innovative gerontological nursing suitable for the fourth industrial revolution era. This phase used Mezirow’s transformative learning theory [23, 24] and a comprehensive literature review, including references to theoretical determinants, to structure the educational program.

#### Theory-based methods and strategies

The program leveraged transformative learning, engaging students through critical reflection and discourse, shifting from transmissive to transformative learning paradigms.

#### Course design

Integrating information from earlier phases, the course was developed with detailed materials and validated for the target population, incorporating aspects of medical technology in gerontological care.

#### Implementation and evaluation

Detailed plans were formulated for program execution, participant engagement, and mediation framework, with the program’s effectiveness evaluated through scientific methods.

### Pilot program intervention

This pilot intervention followed a single-group pretest-posttest design.

#### Participants

Enrolled were 30 individuals from Gyeongsangnam-do, Korea, including both registered hospital nurses and clinical nursing graduate students. Criteria for participation included an understanding of the study’s purpose, willingness to participate, possession of a nursing license, and at least one year of clinical experience.

#### Research measurements

In the pilot program intervention, comprehensive measurements were conducted using validated tools to assess participants’ perspectives and knowledge on gerontological nursing:

##### Ageism and attitudes:

We employed the Fraboni Scale of Ageism (FSA) [25] and Kogan’s Attitudes towards Older People (KAOP) scale [26] to assess ageism and attitudes towards older adults. Both scales have been validated in Korean versions [27, 28] with satisfactory Cronbach’s alpha values.

##### Knowledge assessment:

The Fact on Aging Quiz Part 1 (FAQ 1) by Palmore [29], translated and validated for a Korean context [30], was used to gauge participants’ knowledge about various aging-related aspects.

##### Behavioral change and satisfaction:

To evaluate transformative behavioral changes, a modified Learning Activity Survey [31, 32] was administered, focusing on changes experienced during the learning process. Additionally, program satisfaction was measured using a tailored 5-point Likert scale survey, assessing overall satisfaction, understanding, and usefulness.

#### Education and data analysis

The intensive 8-hour educational program spanned two days, with evaluations conducted using IBM SPSS Statistics 23.0 for pre- and post-program assessments.

## Result

### Phase one - needs assessment

The comprehensive needs assessment involved analyzing the current status and requirements for gerontological nursing education. The assessment included a review of existing literature and consultation with experts in the field to establish a foundational understanding for the program’s development. Through an extensive review of the literature, we pinpointed the prevailing issues faced in providing geriatric nursing education to postgraduate nurses. This examination was crucial for understanding the gaps and areas needing enhancement within the current educational framework. Before moving on to the second phase of our study, which encompassed conducting comprehensive surveys and in-depth interviews, we meticulously crafted and selected specific questions to be included in the survey questionnaire. Additionally, we meticulously formulated questions for the Focus Group Interviews (FGI) to ensure a rich, detailed exploration of the experiences and needs of postgraduate nurses in geriatric care. This preparatory step was fundamental in aligning our data collection instruments with the identified educational challenges and objectives, thereby facilitating a targeted and insightful assessment of the educational needs and preferences of postgraduate nurses in the field of geriatric nursing.

### Phase two - survey and interview

A detailed survey conducted among 154 clinical nurses from 2nd and 3rd tier hospitals in the Gyeongsangnam-do region revealed key demographic and professional data (Table 1). The average age of participants was 36 years, with 55% married. A majority had 1–5 years of total clinical experience (35%), and 58% had less than one year of experience in gerontological care. Approximately 75% held general nurse positions. In terms of gerontological care, 76% believed they were knowledgeable, yet 54.5% had previous experience with related education. The survey highlighted a moderate demand for gerontological nursing education, scoring an average of 3 points, with the highest demand in areas like rehabilitation for older adults (3.4 points), educating older adults on information technology (3 points), and addressing the exclusion of older adults due to medical mechanization (2.9 points).

**Table 1:** General Characteristics of Participants (*N* = 30)

Characteristics	Categories	n (%) or Mean ± SD
Gender	Male	1 (3.3)
	Female	29 (96.7)
Age (years)		33.67 ± 8.30
	20 ~ 29	15 (50)
	30 ~ 39	7 (23.3)
	≥ 40	8 (26.7)
Religion	Christianity	4 (13.3)
	Buddhism	2 (6.7)
	Catholic	1 (3.3)
	Others	1 (3.3)
	No religion	22 (73.3)
Cohabitation experience	Yes	18 (60.0)
	No	12 (40.0)
Relationship (*n* = 18)	Grandparents	13(72.2)
	Parents	3 (16.6)
	One’s parents-in-law	1 (5.6)
	Others	1 (5.6)
Duration of cohabitation (months) (*n* = 18)		69.67 ± 95.40
	< 36	6 (33.3)
	37 ~ 84	3 (16.7)
	> 85	9 (50.0)
Feeling of experience (*n* = 18)	Positive	9 (50.0)
	Negative	4 (22.2)
	Apathy	4 (22.2)
	Others	1 (5.6)
Influence of experience (*n* = 18)	Positive	13(72.2)
	Negative	4 (22.2)
	Apathy	1 (5.6)
	others	0 (21.4)
Volunteer experience	Yes	29 (96.7)
	No	1 (3.3)
Feeling of experience (*n* = 29)	Positive	18(62.1)
	Negative	5 (17.3)
	Apathy	3 (10.3)
	others	3 (10.3)
Influence of experience (*n* = 29)	Positive Negative	26 (89.7) 2 (6.9)
	Apathy Others	1 (3.4) 0 (0.0)
Familiarity with Older Adults	Yea	17(56.7)
	No	13(43.3)
Lectures on gerontological care	Yes	16(53.3)
	No	14(46.7)

Focus group interviews with 14 graduate nursing students from G University identified key educational needs for an innovative gerontological nursing program, emphasizing the importance of character and humanity education, understanding of older adults, technological skills, and enhancing the quality of life for older adults. Preferred educational methods included experiential, simulation-based, and discussion-based learning.

### Phase three - mapping of course objectives

Based on the needs assessment and survey findings, the course objectives were formulated to enable nursing professionals to provide high-quality gerontological care in a rapidly evolving medical environment. Specific objectives targeted improvements in knowledge about older adults and dysphagia, attitudes towards ageism and older adults, and the development of transformative behavior (Table 2).

**Table 2:** Mapping of Course Objectives

Category	Target Items	Description
Cognition	Knowledge About Older Adults and Dysphagia	Enhancing Understanding of Gerontological Aspects and Cognitive Issues in Older Adults.
Attitude	Ageism Attitudes Towards Older Adults Attitudes Towards Caring for Dysphagia	Improving Attitudes Towards Aging in Older Adults and Caring for Dysphagia.
Behavior	Transformative Behavior	Fostering Behavioral Change to Enhance Quality in Gerontological Nursing Practices.

### Phase four - theory-based methods and strategies

The program’s theoretical approach incorporated experiential learning, critical reflection, and rational discourse. Participants experienced the physical challenges of aging through simulation suits and engaged in reflective journal writing to critically assess their perceptions and biases.

### Phase five - course design

The IGNITE program consisted of six targeted modules, each addressing specific learning objectives such as nursing care for geriatric patients with arthritis, prevention of cognitive impairment, and end-of-life care. Innovative educational techniques like simulation and experiential learning were employed to deepen understanding and empathy for older adults. Learning objectives, modules, and teaching methods for the program are presented in Table 3.

**Table 3:** Learning Objectives, Modules, and Teaching Methods in the IGNITE Program

Learning Objectives	Aligned Modules	Teaching Methods
• Learners will be able to describe the normal physiological changes associated with aging and their impact on functional abilities. • Learners will identify common health conditions related to aging and their effects on functional abilities. • Learners will apply person-centered care principles in the nursing care of older adults	Module 1: Introduction to Aging and Functional Changes in the Older Adults	• Watching videos • Lectures
• Learners will conduct a comprehensive nursing assessment for older patients with arthritis, including evaluating pain and functional status. • Learners will create a nursing care plan tailored to the specific needs of older patients with arthritis, focusing on pain management, exercise programs, and pharmacological treatments. • Learners will implement evidence-based nursing interventions to enhance independence, mobility, and quality of life in older arthritis patients.	Module 2: Nursing for Older Adults with Arthritis	• Older adults experience simulation • Reflective journaling • Lectures
• Learners will understand diagnostic methods and nursing care strategies for dysphagia. • Learners will design dietary plans and provide care for individuals with dysphagia. • Learners will undergo a cognitive and emotional transformation regarding dysphagia and craft personal nursing narratives.	Module 3: Nursing for Older Adults with Dysphagia	• Lectures • Group discussions • Presentation with personal insights • Reflective journaling
• Learners will differentiate between mild cognitive impairment and dementia in older adults. • Learners will elucidate a nursing model for improving cognitive functions in older adults. • Learners will boost nursing competence for enhancing cognitive function in older adults with mild cognitive impairment using robotic aids. • Learners will develop and execute strategies to improve cognitive function in older adults with mild cognitive impairment, utilizing robotic aids	Module 4: Preventing Cognitive Impairment	• Lectures • Group discussions • Presentation with personal insights • Reflective journaling
• Learners will define, classify, and explain the symptoms, behaviors, management, and nursing care of dementia. • Learners will comprehend and articulate the definition and four components of person-centered dementia care. • Learners will indirectly experience death and transition cognitively and emotionally.	Module 5: Person-centered care for older persons with dementia	• Lectures • Practicing with assistive care robots • Reflective journaling
• Learners will navigate ethical dilemmas associated with death and develop a new framework for end-of-life care. • Learners will experience a cognitive and emotional shift in end-of-life and pre-dying care, leading to the creation of personal nursing narratives.	Module 6: End of life	• High-fidelity simulation • Reflective journaling • Lectures

### Phase six - implementation and evaluation

The implementation of the IGNITE program began with the recruitment of participants, which was announced through the graduate school bulletin board of G National University. Additionally, cooperation was sought from the head of a nearby hospital to inform potential participants about the program’s content and purpose. The selection criteria for participants included having more than one year of clinical experience, a clear understanding of the study’s purpose, and a voluntary agreement to participate. Due to the COVID-19 pandemic, the group size was restricted to 10 people, and the program was conducted four times, each session spanning two days for a total of 16 h.

The evaluation of the program employed a pre- and post-test design to facilitate a detailed quantitative assessment of its impact. The participants, averaging 34 years in age, mostly had no religious affiliation (73%) and had diverse experiences living with older adults. Key improvements were observed post-program, including a significant decrease in ageism (t = 2.36, *p* =.025) and improved attitudes toward older adult care (t=-4.18, *p* <.001). Additionally, knowledge about dementia and attitudes toward dementia care showed substantial increases. These results are detailed in Table 4.

**Table 4:** The effect of the IGNITE program (*N* = 30)

Variable	Pretest	Posttest	t	p	
	M ± SD	M ± SD			
Ageism	2.16 ± 0.27	2.05 ± 0.29	2.358		0.025
Attitude toward caring for older adults	3.67 ± 0.30	3.89 ± 0.38	-4.181		< 0.001
Knowledge to aging	0.58 ± 0.10	0.59 ± 0.89	− 0.280		0.782
Knowledge to dysphagia	0.75 ± 0.10	0.81 ± 0.73	-3.275		0.003
Attitude toward caring for dysphagia	3.90 ± 0.21	4.17 ± 0.36	-4.265		< 0.001

A major aim of the IGNITE program was to promote transformative behavior. After completing the program, participants reported changes in various aspects of their behavior, with an average transformative behavior score of 7.9 out of 10. This score indicates substantial changes across multiple behavioral aspects.

High levels of participant satisfaction were recorded, with average scores of 4.9 for both program satisfaction and understanding, and 4.8 for usefulness. These satisfaction metrics, along with the detailed process of the program’s development, are presented in Table 5. The research director regularly supervised the interventionists to ensure the quality of the program and addressed operational challenges and sustainable solutions.

**Table 5:** Scores for Transformative Behavior Change and Program Satisfaction among Participants (*N* = 30)

Variable	Range of score	Mean ± SD
Transformative behavior change	1–10	7.93 ± 1.55
Satisfaction with program		
Overall satisfaction with program	1–5	4.88 ± 0.29
Understanding	1–5	4.88 ± 0.29
Usefulness	1–5	4.81 ± 0.40

## Discussion

This study, leveraging the Intervention Mapping Approach (IMA), makes a significant contribution to the field of gerontological nursing education. Notably, it comprehensively encompasses program development, implementation, and evaluation, which marks a departure from the traditional scope of nursing education programs in Korea.

Our study aligns with the emerging trends in nursing education, as highlighted by recent literature. Choi, Lee and Vorderstrasse [33] underscore the necessity for nursing programs to evolve with the changing healthcare landscape, especially with the integration of technological advancements. Our approach, which includes the use of care-assistive robots and simulations, mirrors these recommendations.

The study discusses three important implications. Firstly, it interprets the experiential-critical reflection-rational discourse-action stages as an educational experience in which clinically experienced professional nurses interpret the stages of progressive change. This approach is in line with previous research, which highlights the importance of considering learners holistically and contextually [34]. Transformational learning is a process that creates better beliefs and expectations by changing the referential structure that influences the formation of perception, cognition, and emotion of adult learners, and aims for positive behavior change [35]. In this study, it was confirmed that participants created a new referential structure for resolving cognitive errors they had through writing and sharing their experiences in reflective journals, open discussion, experiential learning, and presentations.

The emphasis on transformative learning in our study finds resonance in the work of Rojo et al. [36], who explored how experiential learning and critical reflection contribute to reshaping the perceptions and practices of nursing professionals. Our findings extend this discussion by demonstrating how nurses can develop new cognitive frameworks that are more inclusive and empathetic towards the needs of older adults, in line with the theoretical determinants identified in our study.

The integration of technology in our educational strategy, particularly the use of medical technology such as care-assistive robots and simulations, is in line with Soriano et al. [37]. This reflects the increasing relevance of technological tools in not only nursing education but also in other health professions like medicine. Their research emphasizes the impact of technology on enhancing the quality and effectiveness of care, especially in gerontological settings.

Our study’s use of the IMA and its focus on mediation within the educational process have broader implications beyond nursing, extending to other health professions. This interdisciplinary approach underscores the importance of integrating various aspects of gerontological care into the curriculum.

The observed behavioral changes in our study participants align with the principles of transition theory, as discussed in Bakon et al. [38]. Their research indicates that educational interventions are pivotal in initiating changes in nursing practices and attitudes. Our findings contribute to this discourse by showing how structured educational programs, based on sound theoretical frameworks, can lead to significant improvements in nursing care for older adults.

The significance of storytelling in our program correlates with the findings of Beierwaltes et al. [39]. They advocate for storytelling as a key strategy in building patient-centered care, particularly in gerontological nursing. Our research extends their findings by demonstrating how storytelling can be effectively utilized to break down barriers, build empathy, and enhance communication skills in nursing students.

This study delves into the broader implications of these educational strategies for the nursing profession and other health professions. It suggests that such innovative approaches are beneficial not only for improving care for older adults but also essential for preparing healthcare professionals across various disciplines to meet the challenges of an increasingly complex healthcare environment.

This study provides valuable insights into the development and efficacy of innovative educational strategies in gerontological nursing. By situating our findings within the wider context of current nursing education research, the study underscores the need for holistic, technologically informed, and person-centered approaches in the care of older adults. Our research contributes to the ongoing dialogue in nursing education, highlighting the imperative for adaptive and empathetic educational models in preparing nursing professionals for the future of healthcare.

## Conclusion

This study significantly contributes to the field of gerontological nursing education through the development and evaluation of the Innovative Gerontological Nursing Intervention Mapping Initiative for Training and Education (IGNITE). This program, designed with a focus on holistic and contextual learning, innovative educational strategies, and person-centered care, demonstrates a substantial potential to enhance nursing practices and outcomes in the care of older adults.

The IGNITE program effectively addressed initial negative perceptions held by clinical nurses towards older adults. Nurses, who initially viewed older adults as burdensome and uncooperative, underwent a transformative learning experience. This process of critical self-reflection and engagement with innovative educational methods led to a paradigm shift in their attitudes and behaviors. The nurses emerged with a newfound empathy, respect, and understanding for older adults, underlining the efficacy of IGNITE in fostering compassionate and empathetic gerontological care.

Furthermore, this study lays a robust theoretical and methodological foundation for the development of adult learning educational materials targeted at clinical nurses. The insights garnered from the IGNITE program can be instrumental in shaping educational content across various healthcare settings, promoting transformative learning and self-reflection. This approach not only leads to innovative nursing practices but also contributes to personal and professional growth in diverse work environments.

Looking ahead, it is imperative to investigate the long-term impacts of the IGNITE program on nursing practices and patient outcomes. Future research should also explore the application of the Intervention Mapping Approach in other healthcare professions and assess the scalability of the IGNITE program. With the global demographic trend towards an aging population, it is crucial to equip nurses and other healthcare professionals with the necessary skills, knowledge, and attitudes to deliver high-quality, person-centered care to this growing vulnerable group.

In conclusion, the IGNITE program represents a significant advancement in gerontological nursing education. It underscores the necessity of ongoing research and development to meet the evolving needs of healthcare professionals in an aging world. This study marks an important step in this direction, highlighting the transformative potential of innovative educational approaches in the realm of nursing and healthcare.

## Data Availability

The data that support the findings of this study are available on request from the corresponding author. The data are not publicly available due to privacy or ethical restrictions.

## Abbreviations

IGNITE: Innovative Gerontological Nursing Intervention Mapping Initiative for Training and Education
FSA: Fraboni Scale of Ageism
K: FAQI-Korean Fact on Aging Quiz Part I
